# Association of rs10811656 on 9P21.3 with the risk of coronary artery disease in a Chinese population

**DOI:** 10.1186/s12944-016-0296-2

**Published:** 2016-08-09

**Authors:** Jianjun Yan, Jinmei Zeng, Zhiyong Xie, Dongchen Liu, Liansheng Wang, Zhong Chen

**Affiliations:** 1 Department of Cardiology, the First Affiliated Hospital of Nanjing Medical University, Nanjing, 210029 China; 2 Devision of Cardiology, The Affiliated Jiangning Hospital of Nanjing Medical University, Nanjing, 211100 China; 3 Department of Cardiology, Shanghai Jiao Tong University Affiliated Sixth People’s Hospital, East Campus, No. 222, Huanhu Xisan Road, Pudong New Area, Shanghai 201306 China

**Keywords:** SNP, single nucleotide polymorphism, Association, rs10811656, CAD, coronary artery disease, Susceptibility

## Abstract

**Background:**

Genome-wide association studies have reported that the 9p21.3 locus confers risk for coronary artery disease (CAD). However, it is not known whether rs10811656 is linked with CAD in a Chinese population. Thus, the purpose of this study was to investigate the potential association between rs10811656 and the risk of CAD in a Chinese population.

**Methods:**

We conducted a hospital-based, case–control study with 251 CAD patients and 304 controls to examine the potential association of rs10811656 with CAD.

**Results:**

The frequencies of the TT genotypes in CAD cases were significantly different from those in controls (adjusted OR: 1.96, 95 % CI: 1.09–3.505, *P* = 0.024). Compared to controls, rs10811656 was significantly associated with the stable angina pectoris (adjusted OR: 1.42, 95 % CI: 1.06–1.90, *P* = 0.017), but not with acute coronary syndrome. There was also a highly significant association of rs10811656 with double-vessel and triple-vessel disease when patients were divided into subgroups based on the number of diseased vessels (adjusted OR: 1.68 and 1.60, 95 % CI: 1.14–2.44 and 1.10–2.33, *P* = 0.009 and 0.02, respectively).

**Conclusion:**

Our results suggest that the rs10811656 locus might be associated with CAD in a Chinese Han population.

## Background

Coronary artery disease (CAD) is one of the major causes of morbidity and mortality throughout the world, including China [1, 2]. Epidemiological studies have identified several risk factors for CAD, including dyslipidaemia, diabetes, obesity, smoking and genetic variance [1]. Most recently, genomics analyses have revealed a series of new candidate markers that may contribute to the pathogenesis of CAD [3].

The 9p21.3 risk locus spans a >50-kb genomic region and comprises 59 linked single nucleotide polymorphisms (SNPs) that are in strong linkage disequilibrium. This genomic region encodes the cyclin-dependent kinase inhibitors, CDKN2A (p16 and p14) and CDKN2B (p15) [4]. Recently, genome-wide association studies have shown that common variants in the 9p21.3 gene desert might be robustly associated with the risk of CAD, acute coronary syndrome (ACS), early atherosclerosis, abdominal aortic aneurysm, and intracranial aneurysm [5–7], although the mechanisms underlying these associations are not completely understood. However, it remains unknown whether rs10811656 polymorphism in this region might be linked with the risk of CAD.

The objective of the present hospital-based, case–control study was to investigate the potential association between rs10811656 and the risk of CAD, and further replicate the positive association of rs10811656 and ACS in a Chinese population.

## Methods

### Study population

This association study consecutively enrolled 251 unrelated consecutive CAD patients, who were all admitted to Shanghai Jiao Tong University Affiliated Sixth People’s Hospital, East Campus from January 2014 to December 2015. CAD was defined as ≥ 50 % luminal narrowing in at least one coronary artery. Patients with CAD were divided into subgroups with 1-vessel, 2-vessel or 3-vessel disease according to the number of significantly affected vessels using the Coronary Artery Surgery Study classification [8]. Two cardiologists who were blinded to all patient information assessed the angiograms. Control subjects (*n* = 304), who underwent coronary angiography to rule out CAD, were also enrolled during the same time period at the same hospital. All subjects enrolled in this study were of Han Chinese origin and had no history of significant concomitant diseases, including cardiomyopathy, bleeding disorders, renal failure, thyroid disease, pulmonary hypertension, and malignancy. This study was approved by the Ethics Committee of Shanghai Jiao Tong University Affiliated Sixth People’s Hospital, East Campus and informed consent was obtained from each participant. All authors had no access to information that could identify individual participants after data collection.

### DNA extraction and genotyping

Peripheral venous blood was drawn from each participant in the first day after admission. Genomic DNA was extracted using the AxyPrep DNA Blood kit (Axygen Scientific Inc, Union City, CA, USA). The rs10811656 polymorphism was genotyped by PCR-LDR sequencing method, as reported previously [9].

### Statistical analysis

The allele frequencies and genotype distributions of the CAD cases and controls were compared using chi-square (*χ*2) tests. Among the controls, the genotype frequencies were tested using the *χ*2-test test for the Hardy-Weinberg equilibrium (HWE). The association between the genotypes and CAD was evaluated by computing odds ratios (ORs) and 95 % confidence intervals (95 % CIs). A multiple logistic regression model was used to adjust for covariates including age, sex, body mass index, smoking status, hypertension, diabetes, and dyslipidemia. The linear trend in the association of rs10811656 polymorphism with CAD severity was evaluated by the *χ*2-test for trend. Two-tailed P values < 0.05 were considered significant. All of the statistical analyses were performed using SPSS for Windows version 13.0 (SPSS, Inc., Chicago, USA).

## Results

### Demographic information

The baseline characteristics of the case and control subjects are presented in Table 1. Patients with CAD were older and had higher rate of smoked and diabetes compared to the control subjects. Based on coronary angiography, 133 (43.75 %) patients in the CAD group had single-vessel disease, 85 (27.97 %) had double-vessel disease and 86 (28.28 %) had triple-vessel disease.

**Table 1:** Baseline characteristics of the study subjects

Characteristic	Controls (*n* = 251)	CAD cases (*n* = 304)	*P* values
Age (years)	63.0 ± 14.5	67.13 ± 11.7	<0.001
Sex (male), n (%)	47 (39.8)	127 (63.8)	<0.001
Hypertension, n (%)	73 (61.9)	141 (71.2)	0.086
Diabetes, n (%)	16 (13.6)	65 (32.7)	<0.001
Dyslipidemia, n (%)	39 (33.1)	76 (38.2)	0.358
Smoking, n (%)	23 (19.5)	71 (35.7)	0.002
TC (mmol/L)	4.57 ± 1.70	4.41 ± 1.26	0.21
TG (mmol/L)	1.94 ± 5.35	1.69 ± 2.02	0.45
HDL-C (mmol/L)	1.18 ± 0.30	1.15 ± 0.27	0.23
LDL-C (mmol/L)	2.81 ± 076	2.69 ± 0.86	0.09
Number of diseased vessels			
One, n (%)	-	133 (43.75 %)	—
Two, n (%)	-	85 (27.97 %)	—
Three n (%)	-	86 (28.28 %)	—

*HDL-C* high density lipoprotein cholesterol, *LDL-C* low density lipoprotein cholesterol, *TC* total cholesterol, *TG* triglyceride

### Genotypes and allele frequencies in CAD cases and controls and their associations with CAD

The minor allele (T) frequency of rs10811656 was 0.3817, and the genotype distribution in our study subjects showed no deviation from the Hardy–Weinberg equilibrium (*P* > 0.05). The distribution of the T genotype was also markedly different between CAD patients and controls (*P* = 0.024), as shown in Table 2. After adjustment for age, sex, hypertension, diabetes, and dyslipidemia, the OR for CAD in subjects with the variant genotypes (CT and TT) was 1.43 (95 % CI: 0.98–2.08, *P* = 0.057). We also found that homozygous TT carriers had a 96 % increase in risk of CAD compared with CC carriers (95 % CI: 1.09–3.505, *P* = 0.024). In the subgroup of patients with stable angina pectoris (SAP), carriers of TT homozygous carriers had a 42 % increase in the risk of CAD compared with the CC carriers (95 % CI: 1.06–1.90, *P* = 0.017). However, no significant association was observed with ACS (Table 3.).

**Table 2:** Distribution of genotypes and the CAD risk estimates for rs10811656

		Controls (*n* = 243)	Cases (*n* = 298)	Crude OR (95 % CI)	*P*	Adjusted^a^ OR (95 % CI)	*P*
	Genotypes, n (%)						
rs10811656	CC	99 (40.7)	103 (34.6)	1.00		1.00	
	CT	118 (48.6)	147 (49.3)	1.19 (0.83–1.72)	0.33	1.32 (0.89–1.94)	0.164
	TT	26 (10.7)	48 (16.1)	1.77 (1.02–3.08))	0.041	1.96 (1.09–3.505)	0.024
	CT + TT	144 (59.3)	195 (65.4)	1.30 (0.92–1.84)	0.14	1.43 (0.98–2.08)	0.057

*CI* confidence interval, *OR* odds ratio

^a^Adjusted for age, sex, body mass index, smoking status, hypertension, diabetes, dyslipidemia

**Table 3:** Association of rs10811656 with SAP and ACS

SNP	Groups	Genotype	Crude OR (95 % CI)	*P*	Adjusted OR (95 % CI)^a^	*P*
rs10811656		CC/CT/TT				
	Control	99/118/26	1		1	
	SAP	66/99/36	1.36 (1.02–1.79)	0.03	1.42 (1.06–1.90)	0.017
	ACS	34/48/12	1.16 (0.81–1.65)	0.41	1.27 (0.87–1.85)	0.21

*ACS* acute coronary syndrome, *CI* confidence interval, *OR* odds ratio, *SAP* stable angina pectoris

^a^Adjusted for age, sex, body mass index, smoking status, hypertension, diabetes, and dyslipidemia

### Stratified analyses of the polymorphism and CAD

Stratified analyses were conducted according to age, gender, and smoking status (Table 4). We noted that T allele was significantly associated with CAD in males (adjusted OR: 1.82, 95 % CI: 1.10–3.01, *P* = 0.02) and smokers (adjusted OR: 2.13, 95 % CI: 1.06–4.26, *P* = 0.033), but not in females (adjusted OR: 1.09, 95 % CI: 0.62–1.91, *P* = NS) or non-smokers (adjusted OR: 1.21, 95 % CI: 0.77–1.89, *P* = NS). Patients with CAD were subclassified into 3 subgroups (single-, double- and triple-vessel disease) based on the number of stenotic coronary arteries. The frequency of patients with T variant genotypes increased from double- to triple-vessel disease, but not with single-vessel disease (Table 5).

**Table 4:** Stratification analysis by age, sex and smoking statue for the association of rs10811656 with CAD

SNPs	Groups	Genotype		Crude OR (95 % CI)	*P*	Adjusted OR (95 % CI)^a^	*P* ^a^
		Case	Control				
rs10811656		CC/CT + TT					
	Age (median)						
	< 66 years	43/88	47/88	1.09 (0.66–1.82)	0.73	1.20 (0.69–2.10)	0.521
	≥ 66 years	60/107	52/56	1.66 (1.01–2.71)	0.045	1.63 (0.98–2.72)	0.061
	Sex						
	Males	63/128	50/61	1.66 (1.03–2.69)	0.037	1.82 (1.10–3.01)	0.02
	Females	49/83	40/67	0.98 (0.58–1.67)	0.967	1.09 (0.62–1.91)	0.76
	Smoking						
	Yes	37/72	27/30	1.75 (0.91–3.37)	0.093	2.13 (1.06–4.26)	0.033
	No	66/123	72/114	1.17 (0.77–1.79)	0.447	1.21 (0.77–1.89)	0.398

MAF rs10811656 T = 38.17 %, P _for HW_ = 0.379793

*CI* confidence interval, *OR* odds ratio

^a^Adjusted for age, sex, body mass index, smoking status, hypertension, diabetes and dyslipidemia

**Table 5:** Association of rs10811656 with the number of diseased vessels

SNP	Group	Genotype	Crude OR (95 % CI)	*P*	Adjusted OR (95 % CI)^a^	*P*
rs10811656		CC/CT/TT				
	Control	99/118/26	1		1	
	One-vessel disease	49/67/14	1.07 (0.78–1.48)	0.67	1.14 (0.82–1.60)	0.42
	Two-vessel disease	25/43/17	1.57 (1.09–227)	0.015	1.68 (1.14–2.44)	0.009
	Three-vessel disease	29/37/17	1.41 (0.97–2.04)	0.068	1.60 (1.10–2.33)	0.02

*CI* confidence interval, *OR* odds ratio

^a^Adjusted for age, sex, body mass index, smoking status, hypertension, diabetes, and dyslipidemia

### Lipid profiles of CAD cases and controls in the rs10811656 genotypes

Table 6 shows lipid profiles according to the rs10811656 genotypes in the CAD and control groups. We did not find a significant association between the rs10811656 genotypes and serum triglycerides, HDL-C or LDL-C levels in these two groups.

**Table 6:** Lipid profiles based on the rs10811656 genotypes in the CAD cases and controls

	Characteristics	Controls		*P*	CAD cases		*P*
SNP		CC	CT + TT		CC	CT + TT	
rs10811656	TG (mmol/L)	1.46 ± 1.08	2.21 ± 6.91	0.294	1.66 ± 1.01	1.70 ± 2.38	0.874
	TC (mmol/L)	4.28 ± 1.11	4.75 ± 2.00	0.039	4.40 ± 1.30	4.40 ± 1.20	0.995
	HDL-C (mmol/L)	1.16 ± 0.27	1.21 ± 0.32	0.235	1.15 ± 0.26	1.16 ± 0.28	0.759
	LDL-C (mmol/L)	2.72 ± 0.79	2.86 ± 0.73	0.164	2.70 ± 0.89	2.67 ± 0.84	0.754

*HDL-C* high density lipoprotein cholesterol, *LDL-C* low density lipoprotein cholesterol, *TC* total cholesterol, *TG* triglycerides

## Discussion

The present case–control study demonstrated that the homozygous TT genotype of rs10811656 on chromosome 9p21.3 increased the risk of CAD in a Chinese population. The 9p21.3 risk locus of the genes encoding the cyclin-dependent kinase inhibitors, CDKN2A (p16 and p14) and CDKN2B (p15), are known as suppressors that control cell cycle and cellular senescence [10, 11]. Moreover, smooth muscle cells homozygous for the 9p21.3 risk alleles showed reduced expression of p16 and p15, thus increasing proliferation and causing failure to enter senescence in several studies [4, 10]. In addition, homozygous carriers of the 9p21 risk allele exhibit upregulation of gene sets involved in cellular proliferation of white blood cells [12].

Harismendy et al. demonstrated that the 9p21.3 locus alters CAD risk by modulating inflammatory signaling through altered STAT1 binding and may modify immune responses by regulating expression of IFN, a 21 or related type I IFN; and this in turn affects the expression of the cyclin-dependent kinase inhibitor gene, CDKN2A/B, which is important regulator of cell proliferation, aging, and apoptosis [13]. Furthermore, in the primary HAoSMCs homozygous TT genotype of rs10811656, reduced expression of p16 and p15disrups TEAD factor binding and TEAD3-dependent transforming growth factor β induction of p16, indicating the TT genotype of rs10811656 could be a functional variant [14].

In the present study, we observed that the distribution of T genotype was also markedly different between CAD patients and controls After adjusting for risk factors including age, sex, smoking status, hypertension, diabetes, and dyslipidemia, the OR for CAD in subjects with the variant genotypes (CT and TT) was 1.43, which was of borderline significance (*P* = 0.057). We also found that homozygous TT carriers had a 96 % increase in the risk of CAD compared with the CC carriers (*P* = 0.024). In addition, TT genotype carriers had a 42 % increased risk of SAP (95 % CI: 1.06–1.90, *P* = 0.017); however, the risk of ACS was not significantly increased. Our results are not consistent with the observations of Qiang et al. [15]. They reported that there was a higher risk of ACS in subjects with than without the rs10811656 T allele [15]. Studies examining the association of disease severity with the 9p21 genotype are conflicting [15, 16]. Anderson et al. and Chen et al. found that the 9p21.3 allele predicted the prevelance of CAD, but it did not predict its severity [16, 17]. The investigation by Muendlein et al. showed that the variant rs1333049 was associated with angiographically characterized coronary atherosclerosis instead of plaque instability, rupture, and thrombogenesis [18]. However, in a study of 769 patients, Ye et al. demonstrated that 9p21 is associated with a higher prevalence and 5-year progression of preclinical atherosclerosis in the carotid vessels, suggesting that 9p21 promotes plaque formation [19]. Although we did not observe a significant relationship between the rs10811656 T allele and ACS, we found that the frequency of patients with T-variant genotypes increased from double- to triple-vessel disease, whereas there was no association with single-vessel disease. Buysschaert et al. demonstrated that certain alleles on the 9p21 locus are associated with a predisposition to recurrent myocardial infarction and cardiac death within 6 months after the primary event [20].

Associations of SNPs on chromosome 9p21.3 with cardiovascular disease may reflect an influence on the development and progression of coronary atherosclerosis in native arteries rather than causing acute rupture of vulnerable plaques and thrombosis. Cheng et al. found that rs10811661 on 9p21.3, as the neighbor SNP of rs10811656, was associated with type 2 diabetes and CAD, but it was not correlated with the severity of coronary atherosclerosis in a Chinese Han population [21]. The results of these previous studies taken together with our results suggest that further investigation is warranted to determine whether the CAD risk alleles on 9p21.3 are related to the severity of coronary atherosclerosis.

Our stratification analysis showed that the T allele was significantly associated with CAD in males. Males who carried the variant genotypes (TT + TC) had a significantly higher risk of CAD than the CC carriers. The T allele could be a genetic susceptibility marker for CAD in males. Valentina et al., reported that in males with breast cancer, the rs1011970/9p21.3 risk genotype was associated with HER2+ disease [22]. Lin et al. reported that chromosome 9p21 had a significant association with carotid atherosclerosis, especially the intima-media thickness of the internal carotid artery, in a gender-specific manner in a Chinese Han population [23]. However, little is known regarding the functional consequences of variants on 9p21.3, or why the variants exert a gender-specific effect on the risk of CAD. Further research will be needed to elucidate the biological significance of these associations.

We noted that among smokers, carriers of homozygous TT and heterozygous TC had an increased risk of CAD than CC genotype carriers. Our result suggests that the rs10811656 polymorphism may be factor that contributes to smoking-related CAD in a Chinese population. There was also no significant association between the rs10811656 genotypes and serum triglycerides, HDL-C and LDL-C levels in the CAD patients and controls. Therefore, our study revealed that the risk allele was associated with angiographically characterized coronary atherosclerosis, suggesting that the risk SNP exerts cell or tissue-specific effects relevant to atherosclerosis rather than affecting the traditional risk factors for CAD.

Our study has several limitations. First, selection bias and a relatively small sample size in the present study may have reduced the statistical power of the results, even though the genotype distribution of patients and controls in our study was compatible with the Hardy–Weinberg expectations. Secondly, a prospective long-term outcome study is warranted to analyze the association of polymorphism with long-term prognosis in CAD patients. Lastly, our data should be extrapolated to other regions and ethnic groups cautiously.

## Conclusion

To the best of our knowledge, the present study demonstrates for the first time that the rs10811656 polymorphism is associated with increased risk of CAD in a Chinese population. Additionally, in male subjects and smokers, rs10811656 homozygous carriers of TT have an increased risk of CAD. Moreover, we found that the rs10811656 T variant is significantly correlated with SAP and angiographically characterized coronary disease severity. In order to enhance understanding of the relationship between this polymorphism and risk of CAD, further large-scale studies are required in other populations.
